# Post-Marketing Surveillance of Human Rabies Diploid Cell Vaccine (Imovax) in the Vaccine Adverse Event Reporting System (VAERS) in the United States, 1990‒2015

**DOI:** 10.1371/journal.pntd.0004846

**Published:** 2016-07-13

**Authors:** Pedro L. Moro, Emily Jane Woo, Wendy Paul, Paige Lewis, Brett W. Petersen, Maria Cano

**Affiliations:** 1 Immunization Safety Office, Division of Healthcare Quality Promotion (DHQP), National Center for Zoonotic and Emerging Infectious Diseases (NCZEID), Centers for Disease Control and Prevention (CDC), Atlanta, Georgia, United States of America; 2 Center for Biologics Evaluation and Research, Food and Drug Administration, Silver Spring, Maryland, United States of America; 3 Poxvirus and Rabies Branch, Division of High-Consequence Pathogens and Pathology, NCZEID, CDC, Atlanta, Georgia, United States of America; Naval Medical Research Center, UNITED STATES

## Abstract

**Background:**

In 1980, human diploid cell vaccine (HDCV, Imovax Rabies, Sanofi Pasteur), was licensed for use in the United States.

**Objective:**

To assess adverse events (AEs) after HDCV reported to the US Vaccine Adverse Event Reporting System (VAERS), a spontaneous reporting surveillance system.

**Methods:**

We searched VAERS for US reports after HDCV among persons vaccinated from January 1, 1990–July 31, 2015. Medical records were requested for reports classified as serious (death, hospitalization, prolonged hospitalization, disability, life-threatening-illness), and those suggesting anaphylaxis and Guillain-Barré syndrome (GBS). Physicians reviewed available information and assigned a primary clinical category to each report using MedDRA system organ classes. Empirical Bayesian (EB) data mining was used to identify disproportional AE reporting after HDCV.

**Results:**

VAERS received 1,611 reports after HDCV; 93 (5.8%) were serious. Among all reports, the three most common AEs included pyrexia (18.2%), headache (17.9%), and nausea (16.5%). Among serious reports, four deaths appeared to be unrelated to vaccination.

**Conclusions:**

This 25-year review of VAERS did not identify new or unexpected AEs after HDCV. The vast majority of AEs were non-serious. Injection site reactions, hypersensitivity reactions, and non-specific constitutional symptoms were most frequently reported, similar to findings in pre-licensure studies.

## Author Summary

In 1980, human diploid cell rabies vaccine (HDCV, Imovax Rabies, Sanofi Pasteur), was licensed for use in the United States. To assess adverse events (AEs) after HDCV reported to the US Vaccine Adverse Event Reporting System (VAERS), a spontaneous reporting surveillance system. We searched VAERS for US reports after HDCV among persons vaccinated from January 1, 1990–July 31, 2015. Medical records were requested for reports classified as serious (death, hospitalization, prolonged hospitalization, disability, life-threatening-illness), and those suggesting anaphylaxis and Guillain-Barré syndrome (GBS). Physicians reviewed available information and assigned a primary clinical category to each report using the Medical Dictionary for Regulatory Activities (MedDRA) system organ classes. We used a special type of analysis, Empirical Bayesian data mining, to identify AEs reported more frequently after HDCV than the same AE after other vaccines. VAERS received 1,611 reports after HDCV;93 (5.8%) were serious. Among all reports, the three most common AEs included pyrexia (18.2%), headache (17.9%), and nausea (16.5%). Among serious reports, four deaths appeared to be unrelated to vaccination. This 25-year review of VAERS did not identify new or unexpected AEs after HDCV. The vast majority of AEs were non-serious. Injection site reactions, hypersensitivity reactions, and non-specific constitutional symptoms were most frequently reported, similar to findings in pre-licensure studies.

## Introduction

Three cell culture rabies vaccines are licensed in the United States: human diploid cell vaccine (HDCV, Imovax Rabies, Sanofi Pasteur), purified chick embryo cell vaccine (PCECV, RabAvert, Novartis Vaccines and Diagnostics), and rabies vaccine adsorbed (RVA, Bioport Corporation). Only HDCV and PCECV are available for use in the United States [1]. These vaccines are indicated for post- and pre-exposure prophylaxis to prevent human rabies [1,2,3,5]. Rabies post-exposure prophylaxis (PEP) involves prompt and thorough wound cleansing followed by passive immunization with human rabies immunoglobulins (HRIG) and vaccination with four doses of HDCV or PCECV (given in a series separated by several days) for persons previously unvaccinated with rabies vaccine (five doses in persons with altered immunocompetence). Persons who previously received a complete vaccination series (pre-exposure or postexposure) should receive only two doses of vaccine. Pre-exposure vaccination, with three doses of either vaccine given for a primary course, is recommended for persons in high-risk groups such as veterinarians and their staff, animal handlers, rabies researchers, and certain laboratory workers. Pre-exposure vaccination should also be considered in persons (e.g., international travelers) who are likely to come in contact with rabid animals in areas or countries where dog or other animal rabies is enzootic and immediate access to appropriate medical care, including rabies vaccine and immune globulin, might be limited [1,2]. Serologic monitoring of vaccinated persons in the highest risk groups is recommended with a single booster dose of vaccine given if the serum titer falls below the recommended cut off. PCECV became available in 1997 [3] and a safety assessment of VAERS reports during 1997–2005 was conducted in 2006 [4]. HDCV, which was licensed on June 9, 1980, is prepared from the Pitman-Moore strain of rabies virus grown on MRC-5 human diploid cell culture [5]. In pre-licensure studies of HDCV, local reactions (e.g., pain at the injection site, redness, swelling, and induration) were the most common adverse events (AEs) following vaccination [1], affecting approximately 25% of recipients [5]. Mild constitutional symptoms (e.g., fever, headache, dizziness, and gastrointestinal symptoms) were observed in 20–56% of recipients [1,5]. In one study, up to 6% of persons presented with systemic hypersensitivity reactions after receiving booster vaccination with HDCV following primary rabies prophylaxis, 3% occurring within 1 day of receiving boosters, and 3% occurring 6–14 days after boosters [1,7]. Post-marketing reports of hypersensitivity reactions after HDCV have previously been reviewed [6], but this is the first summary of all AEs reported to VAERS since product licensure more than 30 years ago. In the present study, we assess the safety of HDCV from the inception of VAERS through July 31, 2015.

## Materials and Methods

### Vaccine Adverse Events Reporting System (VAERS)

Established in 1990, VAERS is a national vaccine safety surveillance system, co-administered by the Centers for Disease Control and Prevention (CDC) and Food and Drug Administration (FDA) that receives spontaneous reports (also known as passive surveillance) of AEs following immunization [8]. Anyone can report and adverse event (AE) including healthcare providers, vaccine recipients, vaccine manufacturers, and other reporters. Reports are submitted voluntarily either directly from individual reporters, who may be reporting for themselves or others, or secondarily from vaccine manufacturers, that also receive spontaneous reports and in turn submit them to VAERS. Reporting is encouraged for any clinically important or unexpected AE, even if the reporter is not sure if a vaccine caused the event [8]. VAERS accepts all reports without rendering judgment on clinical importance or whether vaccine(s) might have caused the AE. The VAERS report form collects information on age, sex, vaccine(s) administered, AE(s) experienced, medical conditions at the time of vaccination, and medical history. Signs and symptoms of AEs are coded by trained personnel and entered into a database using the Medical Dictionary for Regulatory Activities (MedDRA), a clinically validated, internationally standardized medical terminology [9]. A VAERS report may be assigned one or more MedDRA preferred terms (PT). A PT is a distinct descriptor for a symptom, sign, disease, diagnosis, therapeutic indication, investigation, surgical, or medical procedure, and medical, social, or family history characteristic [10]. MedDRA PTs are not confirmed diagnoses. Reports are classified as serious based on the Code of Federal Regulations if one of the following is reported: death, life-threatening illness, hospitalization or prolongation of hospitalization, permanent disability, or a congenital anomaly [11]. For serious reports from sources other than vaccine manufacturers, medical records are routinely requested and made available to VAERS personnel. Reports of medication errors (e.g., drug administered to patient of inappropriate age) may also be reported and are assigned MedDRA PTs, even if there is no AE *per se*.

We analyzed US VAERS reports received by July 31, 2015 for persons vaccinated with HDCV during January 1, 1990 through July 31, 2015. We excluded non-US reports and duplicate reports. All patient medical data was anonymized. Because VAERS is a routine surveillance program conducted for public health, it does not meet the definition of research and is not subject to Institutional Review Board approval and informed consent requirements.

### Clinical review of reports

Physicians reviewed all reports and all available medical records for serious reports and the following conditions (regardless of seriousness): anaphylaxis, Guillain-Barré Syndrome (GBS), and pregnancy. The main diagnosis was categorized into a MedDRA System Organ Class (SOC) group. Reports suggestive of anaphylaxis or GBS were verified using the Brighton Collaboration criteria or a physician’s diagnosis [12,13].

### Data mining

We used Empirical Bayesian (EB) data mining [14] to identify AEs reported more frequently than expected following HDCV in the VAERS database. EB data mining screens for vaccine-event pairs that are reported more frequently than expected, i.e. disproportional reporting. Furthermore, EB data mining can minimize false-positive signals resulting from the algorithm’s shrinkage towards the null when observed and/or expected counts are low. EB05 is defined as the lower 90% CI limit of the adjusted ratios of the observed counts over expected counts [15]. Through data-mining analysis, HDCV reports were compared with all other vaccines in the VAERS database. We used published criteria [15,16] to identify, with a high degree of confidence, HDCV vaccine-event pairs reported at least twice as frequently as would be expected (i.e., lower bound of the 90% confidence interval surrounding the EB geometric mean [EB05] ≥2). We clinically reviewed those HDCV reports containing PTs which exceeded the data mining threshold noted above.

## Results

**Fig 1:**
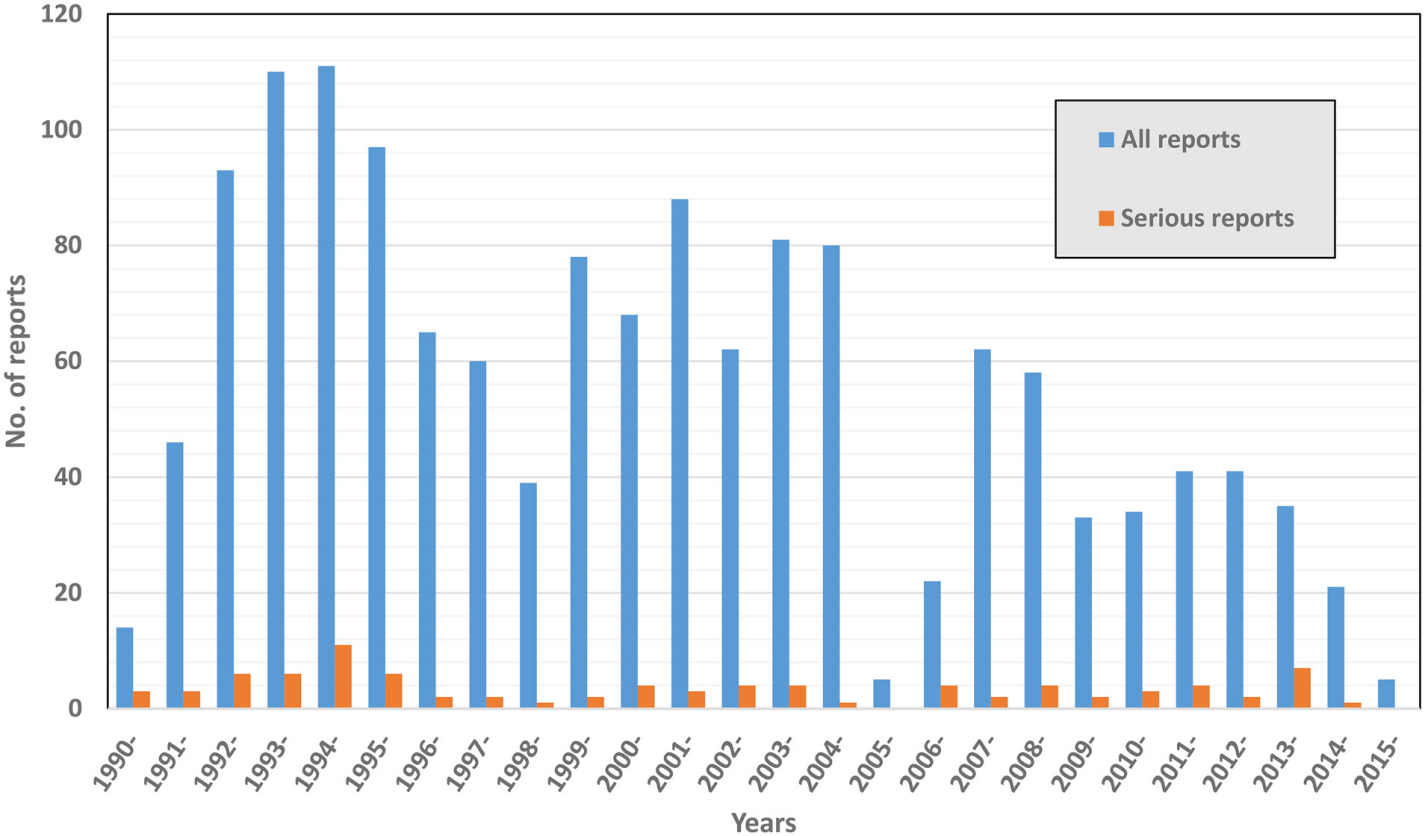
Adverse events after HDCV (Imovax) reported to VAERS, 1990–2015. *162 reports did not have vaccination year provided. Six were serious reports. 2015 is a partial year.

During the 25 years of this study, VAERS received 1,611 reports after HDCV (Table 1); ninety-three (5.8%) were coded as serious which included five deaths (Table 2). In 31 (33.3%) of 93 serious reports, the primary diagnosis was verified by review of medical records. Most reports after HDCV were received during 1992–1996 and 1999–2004. A marked reduction in reports was observed for years 2005 and 2006 (Fig 1). HDCV was the only vaccine listed in 1,424 (88.4%) reports. The three most frequent MedDRA PTs among all reports were pyrexia (293,18.2%), headache (288,17.9%), and nausea (266,16.5%). These PTs were also predominantly noted among serious reports (Table 3).

**Table 1:** Characteristics of human diploid cell vaccine (HDCV, Imovax) rabies vaccine reports to VAERS among persons vaccinated January 1, 1990 through July 31, 2015.

Characteristics	No. (%)
Total Reports	1,611
Serious	93 (5.8)
Female^a^	996 (61.8)
Median onset interval (range) days^b^	1 (0–1095)
Reports where HDCV was the only vaccine listed^c^	1,424 (88.4)
Type of reporter	
Vaccine provider	728 (45.2)
Manufacturer	399 (24.8)
Other	264 (16.4)
Patient	103 (6.4)
Median age (range) years^d^	32 (0–90)
Age groups (years)	
< 18	174 (10.8)
18–29	451 (28.0)
30–39	330 (20.5)
40–49	289 (17.9)
50–59	136 (8.4)
≥ 60	57 (3.5)

^a^Gender not reported in 64 (4.0%) reports.

^b^Onset interval (the time between vaccination and the reported onset of symptoms) was not reported in 146 (10.1%) reports.

^c^Other vaccines given concomitantly included: Typhoid vi Polysacharide in 26 (13.9%), Td adsorbed in 24 (12.8%), and Japanese Encephalitis in 17 (9.1).

^d^Age not reported in 174 (10.8%) reports.

**Table 2:** Reports of deaths following human diploid cell rabies vaccine, VAERS 1990–2015.

Age	Gender	Medical history	Vaccines co-administered with HDCV	Onset interval (days)	Cause of death/circumstances around death
42	F	Non-contributory	Hepatitis B vaccine	5	Pericardial tamponade due to thrombotic thrombocytopenia and hemolytic anemia
74	M	COPD, arteriosclerotic cardiovascular disease	None	2	Complications of dehydration.
37	M	Bitten by bat day before vaccination	None	5	Fatty liver and Mallory Weiss Syndrome consistent with chronic alcoholism
34	F	Depression, gastrointestinal reflux; treated with antibiotics for bronchitis at time of vaccination	None	2	Acute demyelinating encephalomyelitis (ADEM) following rabies vaccination^†^
24	F	Arterial hypertension, no illness at time of vaccination	None	1	Probable cause of death: hypertensive and ischemic heart disease

^†^PCR for rabies and other viruses were negative.

**Table 3:** Most frequent MedDRA PT codes after rabies vaccine (HDCV, Imovax) in serious^†^ and non-serious reports in VAERS among person vaccinated January 1, 1990 through July 31, 201.

	Serious	Non-serious	Total
	(n = 93)	(n = 1,518)	(n = 1,611)
MedDRA Preferred Term*	n (%)		
Headache	20 (21.5)	268 (17.7)	288 (17.9)
Nausea	16 (17.2)	250 (16.5)	266 (16.5)
Vomiting	16 (17.2)	132 (8.7)	148 (9.2)
Pyrexia	15 (16.1)	278 (18.3)	293 (18.2)
Asthenia	14 (15.1)	92 (6.1)	106 (6.6)
Myalgia	14 (15.1)	178 (11.7)	192 (11.9)
Paraesthesia	14 (15.1)	109 (7.2)	123 (7.6)
Urticaria	13 (14.0)	239 (15.7)	252 (15.6)
Dizziness	13 (14.0)	148 (9.8)	161 (9.9)
Arthralgia	12 (12.9)	130 (8.6)	142 (8.8)
Pruritus	11 (11.8)	196 (12.9)	207 (12.8)
Rash	10 (10.8)	122 (8.0)	132 (8.2)
Diarrhea	9 (9.7)	64 (4.2)	73 (4.5)

^†^ Reports are classified as serious based on the Code of Federal Regulations if one of the following is reported: death, life-threatening illness, hospitalization or prolongation of hospitalization, permanent disability, or a congenital anomaly [11].

*One report may contain more than one PT name; therefore percentages may sum to greater than 100%.

### Death reports

There were five death reports after HDCV and the causes of death are shown in Table 2. The causes of death in four reports were unrelated to vaccination. In one report in which the cause of death was acute disseminated encephalomyelitis (ADEM), the possibility that HDCV could have contributed to the condition could not be ruled out. This report involved a 34 year-old female who received two doses of HDCV, 7 days apart. She had received rabies vaccines in the past without problems. Two days after the second dose of HDCV, she presented with hemiparesis, fever, headache, neck pain, photophobia, speech impairment, right leg tremors, nausea and vomiting. She was hospitalized and was treated with steroids and intravenous antibiotics, but her condition worsened and she died 9 days after vaccination. The autopsy found the cause of death as acute disseminated encephalomyelitis (ADEM) following rabies vaccine. Pathological examination of the patient’s brain tissue found pathological changes compatible with ADEM. PCR testing of the same lot of Imovax administered to the patient was negative for rabies virus.

### Non-death serious reports

The most frequent AE diagnostic category noted among non-death serious reports was immune system disorders, which accounted for 23 (26.1%) of 88 reports (Table 4). Sixteen of these reports were hypersensitivity or non-anaphylactic allergic reactions, and seven were reports of anaphylaxis. General disorders and administration site conditions, comprised mainly of constitutional signs and symptoms (e.g., headache, fever), accounted for 21 (23.9%) reports. Nervous system disorders were noted in 18 (20.4%) reports, including GBS (four reports) and seizures (three reports).

**Table 4:** Diagnostic categories for non-death serious reports of adverse events after human diploid cell rabies vaccine (HDCV, Imovax) vaccine in VAERS among persons vaccinated January 1, 1990 through July 31, 2015.

Diagnostic category	HDCV (N = 88)
	N (%)
Immune system disorders	23 (26.1)
Non-anaphylaxis allergic reactions	16
Anaphylaxis^a^	7
General disorders and administration site conditions	21 (23.9)
Nervous system disorders	18 (20.4)
Guillain-Barré Syndrome	4
Seizures	3
Bell’s palsy	1
Other^b^	10
Gastrointestinal disorders	4 (4.5)
Respiratory, thoracic and mediastinal disorders	3 (3.4)
Infections and Infestations	3 (3.4)
Blood and lymphatic system disorders	3 (3.4)
Psychiatric disorders	3 (3.4)
Cardiac disorders	2 (2.3)
Vascular disorders	2 (2.3)
Musculoskeletal and connective tissue disorders	2 (2.3)
Skin and subcutaneous disorders	1 (1.1)
Renal and urinary disorders	1 (1.1)
Injury, poisoning and procedural complications (fall and head injury)	1 (1.1)
None	1 (1.1)

^a^Include two reports Brighton level 1; medical records were not available for the other five reports and level criteria could not be assessed.

^b^Include one report each of: myelopathy/plexopathy/polyneuropathy; nonspecific central nervous disorder; paresthesia/transient ischemic attack versus reaction to vaccine; cervical radiculopathy; multiple neurological symptoms (e.g., twitching, tingling, numbness); chronic inflammatory demyelinating polyradiculopathy, neuropathy generalized numbness/tingling; mild transient ataxia and vasovagal syncope; mental status changes.

### Pre-specified conditions

#### Anaphylaxis

Twelve anaphylaxis reports were identified, seven of which were serious. The onset interval from vaccination to the reported occurrence of symptoms was <24 hours for ten patients. For two patients, the interval was 26 hours and 2 days, respectively. Among the 12 cases, anaphylaxis reportedly occurred after the following doses of HDCV: first dose (1 case), second dose (4), third dose (4), booster (2 for pre-exposure prophylaxis), and not reported (1). Two patients received both PCECV and HDCV; one report stated that anaphylactic reactions occurred after the third dose with PCECV and after the fourth dose with HDCV, and the other report did not specify. One person received concomitant Tetanus and diphtheria toxoids adsorbed vaccine, but the other eleven reports listed HDCV as the only vaccine administered that day. In addition to HDCV, two patients received HRIG concomitantly. Among the serious cases, six recovered, and in one case the recovery status was not reported. Median age was 41 years (range 13–51 years); six reports were in females, four in males and in two, the sex was not reported. Two reports met Brighton level 1 criteria. The other reports could not be further assessed, as no medical records were available for review. Of the five non-serious cases, four reportedly recovered and the recovery status for the other one was not reported. Of note, three of the 12 patients had experienced other adverse events after previous or subsequent doses of rabies vaccine, such as fever, headache, pruritus, angioedema, and rash.

#### Guillain-Barré Syndrome (GBS)

Four reports of GBS after HDCV were received: two in males aged 25 and 42 years, and two in females aged 16 and 47 years. Two reports were verified and both met Brighton level 2 criteria; their time interval from vaccination to onset of symptoms was 12 and 85 days. Among the four GBS patients the 16-year-old female had presented with vomiting and diarrhea 1 week before receiving HDCV. No other patient had a history of gastrointestinal or upper respiratory infections before being vaccinated. All GBS patients presented varying neurological symptoms characterized mainly by increasing weakness, numbness, and/or tingling in extremities and difficulty walking. The 25-year-old male presented with respiratory failure and required intubation. He was treated with intravenous steroids and intravenous immunoglobulin and recovered. Three of the patients recovered, but the recovery status of the fourth was not reported.

#### Pregnancy

Four reports described HDCV vaccination during pregnancy: three during the first trimester, and in the other report the trimester was not reported. AEs noted included two reports of spontaneous abortion, and one each of syncope and a non-anaphylaxis allergic reaction.

### Datamining analysis

Through July 20, 2015, data mining analysis revealed disproportional reporting for the PTs angioedema and urticaria for HDCV. Because these allergic-type signs and symptoms had been reported in the past, no further review was needed. No disproportional reporting was observed for other PTs, including GBS or other neurological conditions.

## Discussion

During the period of the study, a total of 3.8 million doses of HDCV were distributed in the US (data shown with permission of Sanofi Pasteur). In our review of 25 years’ of AE reports to VAERS following HDCV we did not observe any new or unexpected AEs. The vast majority of AEs were non-serious and already known to be associated with HDCV. We noted a marked decrease in the number of reports during 2005 and 2006 which coincides with the recall of several vaccine lots of HDCV during 2004 [17]. The most common AEs reported were consistent with injection site reactions observed during pre-licensure trials and with hypersensitivity reactions that were previously described [1,6]. We also noted that constitutional symptoms (e.g., headache, nausea, fever) were among the most frequently reported AEs, which is consistent with findings from pre-licensure studies [1,5].

During the first four years of post-marketing experience, systemic allergic reactions were observed at a rate of 11 cases per 10,000 vaccinees [6]. Most of these reactions were associated with booster immunizations. Consistent with this earlier finding, we noted (through automated analysis of reports, clinical review of serious reports, and Empirical Bayesian data mining) that hypersensitivity reactions (e.g., urticaria, pruritus) were frequently reported. Anaphylactic reactions were more likely to occur among individuals who had experienced symptoms or signs compatible with a hypersensitivity reaction after HDCV. Although these anaphylactic reactions after HDCV are rare, they pose a serious dilemma for the patient and the attending physician. A patient’s risk for acquiring rabies must be carefully considered before deciding to discontinue vaccination [1,2]. Recommendations from the Advisory Committee on Immunization Practices state that once initiated, rabies prophylaxis should not be interrupted or discontinued because of local or mild systemic adverse reactions to rabies vaccine. Usually such reactions can be successfully managed with anti-inflammatory, antihistaminic, and antipyretic agents. When a person with a history of hypersensitivity to rabies vaccine must be revaccinated, empiric intervention such as pretreatment with antihistamines might be considered. Epinephrine should be readily available to counteract anaphylactic reactions, and the patient should be observed carefully immediately after vaccination [1].

GBS is an acute, immune-mediated paralytic disorder of the peripheral nervous system [18]. GBS is most commonly associated with *Campylobacter jejuni* and other infectious agents, and it is believed to be an autoimmune process triggered by antigenic stimulation leading to demyelination and destruction of peripheral nerves [18]. An increased risk of GBS after influenza vaccination was first observed with the 1976–1977 A/New Jersey (‘‘swine influenza”) vaccine [19]. Although questions about GBS following non-influenza vaccines have been raised, no study has confirmed a causal association between GBS and other vaccinations [20].

Acute disseminated encephalomyelitis (ADEM) was noted as the cause of death in one report. ADEM is a severe and sudden demyelinating disease which may occur following viral or bacterial infections, and less often, following vaccinations [21]. However, the association between ADEM and vaccination, including rabies vaccines, are mostly from case reports [22–24]. The Institute of Medicine reviewed the available evidence for a limited number of vaccines (not including rabies vaccines) and found there was no evidence to accept or reject a causal association between ADEM and vaccination [25].

VAERS is a national system which is useful for identifying rare AEs and events that were not observed during pre-licensure trials. Any safety concern or ‘signal’ should be studied in other systems or through the design of epidemiological studies [8]. VAERS is a spontaneous reporting system that has important limitations which include over- or under-reporting, biased reporting, and inconsistency in quality and completeness of reports [8]. With few exceptions, such as injection site reactions, VAERS generally cannot assess causality between an AE and receipt of a vaccine.

Rabies is a life-threatening disease, and the benefits of vaccination far outweigh the risks in persons exposed or potentially exposed to the virus. Our findings are reassuring. Most AEs were non-serious and have previously been described.

## Supporting Information

S1 Checklist (PDF)
